# Flecainide-Induced Left Bundle Branch Block

**DOI:** 10.7759/cureus.24385

**Published:** 2022-04-22

**Authors:** Swetha R Nuthulaganti, Yixin Zhang, Temitope Akinjogbin, Khadeeja Esmail

**Affiliations:** 1 Internal Medicine, University of Florida College of Medicine – Jacksonville, Jacksonville, USA; 2 Cardiology, University of Florida College of Medicine – Jacksonville, Jacksonville, USA; 3 Cardiology, University of Florida College of Medicine - Jacksonville, Jacksonville, USA

**Keywords:** acute kidney injury, flecainide, ekg abnormalities, left bundle branch block (lbbb), atrial fibrillation (af)

## Abstract

Flecainide is the first-line antiarrhythmic agent used in patients without structural heart disease. It is a class IC antiarrhythmic drug that inhibits inward sodium current through its dose-dependent blockade of voltage-gated calcium channels within the cardiac membrane. It has been shown to slow the conduction in the left bundle branch block. Unmonitored toxicity can cause ventricular dyssynchrony or fatal arrhythmia. We present a case in which flecainide use caused a new left bundle branch block (LBBB).

## Introduction

Atrial fibrillation is a common arrhythmia seen primarily in adult patients with several comorbidities. Risk factors for atrial fibrillation include metabolic syndrome, hyperthyroidism, excessive intake of alcohol, sepsis, electrolyte abnormalities, etc. Treatment options in patients with atrial fibrillation vary based on symptoms, cardiac structure and function, and the presence of systolic heart failure.

When deciding treatment options for atrial fibrillation two options of medical management are available: rate control and rhythm control. In patients with concern for decompensated heart failure, rhythm control is preferred. In patients with co-existing heart failure, atrial fibrillation is often poorly tolerated and can increase the risk of decompensation and worsening cardiomyopathy [1]. Rhythm control can be achieved through medications (flecainide, sotalol, amiodarone, etc.), electrical cardioversion, and/or catheter ablation. There are four classes of antiarrhythmics - Cass I: voltage-gated sodium channel modulators (quinidine, disopyramide, lidocaine, flecainide), Class II: autonomic modulators (beta-blockers, beta-agonists, muscarinic blockers, adenosine agents), Class III: potassium channel modulators (amiodarone, sotalol), Class IV: calcium channel modulators autonomic modulators (L-type calcium channel blockers). For the patient noted in the case presentation, flecainide was the agent used for rhythm control.

## Case presentation

**Figure 1:**
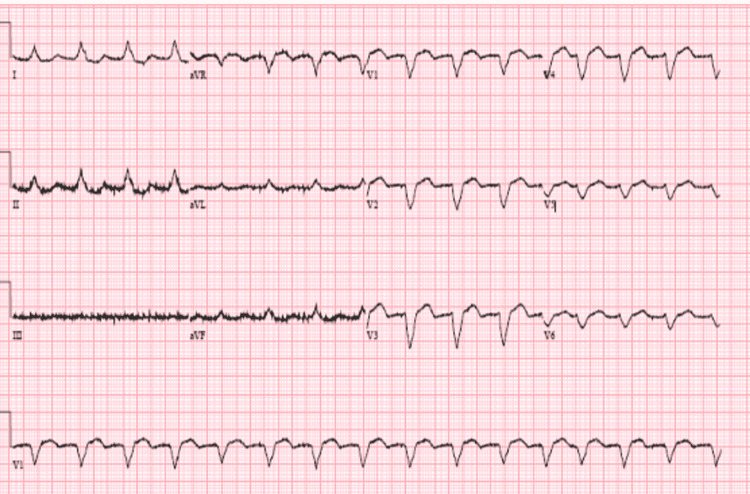
Left bundle branch block caused by flecainide toxicity

**Figure 2:**
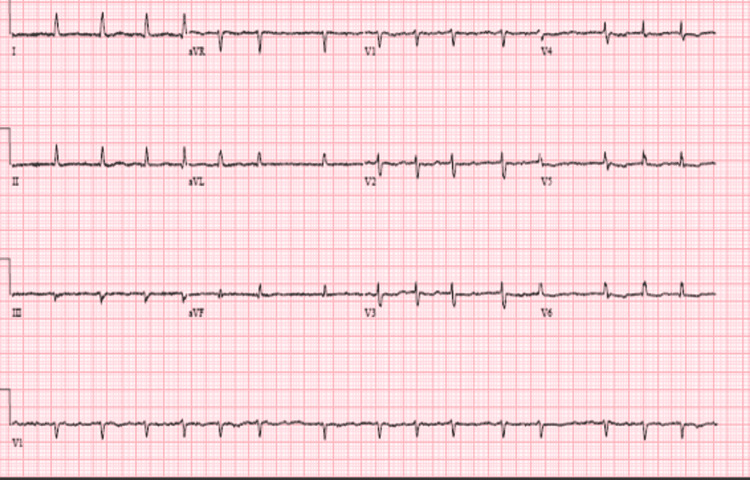
Improvement of left bundle branch block following the discontinuation of flecainide

A 57-year-old female with a past medical history of hypertension, heart failure with a preserved ejection fraction of 55% to 60%, paroxysmal atrial fibrillation status post cardioversion (on Xarelto and flecainide), and hypothyroidism, was admitted following a motor vehicle accident. Upon admission, initial vitals were significant for a blood pressure of 140/98 mmHg, heart rate (HR) of 117 beats per minute, respiration of 20 respirations per minute, and oxygen saturation of 99% on room air. A computed tomography angiography (CTA) of trauma to the thoracic aorta demonstrated right breast lesions suggestive of hematoma, and large right pleural effusion. Arm X-rays demonstrated distal radial fracture with dorsal angulation, and comminuted distal tibial and fibular fractures with lateral displacement. A chest X-ray demonstrated cardiomegaly, and a trace right pleural effusion versus right subsegmental atelectasis was seen on CT as well. Laboratory results were unremarkable. An electrocardiogram (EKG) demonstrated normal sinus rhythm. The remainder of the labs were within normal limits. The patient was scheduled for external fixation of the left ankle and support non-operative management for the right distal radius. Twelve days following the external fixation of the left ankle, the patient was taken back to the operation room for removal of external fixation and definite fixation of the left ankle. Following surgery, the patient was noted to have an acute kidney injury with a blood urea nitrogen (BUN) of 43 and a creatinine of 2.50mg/dL (baseline 0.99). The patient’s EKG was notable for sinus rhythm, HR of 96bpm with a new left bundle branch block (LBBB) (Figure 1). The patient was asymptomatic and hemodynamically stable. A repeat echocardiogram was completed that confirmed preserved ejection fraction of 60% to 65% without any evidence of wall motion abnormalities. The new LBBB was thought to be caused by the flecainide. Once the flecainide was discontinued, the patient reverted back to atrial fibrillation; however, there was an immediate resolution of the LBBB (Figure 2). The patient was then switched to diltiazem for rate control for atrial fibrillation. Outpatient cardiology follow-up was scheduled at discharge. 

## Discussion

Flecainide, much like the other antiarrhythmics, has membrane-stabilizing activity. It is the first-line antiarrhythmic in patients with atrial fibrillation without structural heart disease [2]. As a class IC antiarrhythmic drug, flecainide inhibits inward sodium current through its dose-dependent blockade of voltage-gated calcium channels within the cardiac membrane [3]. By doing so, flecainide ultimately slows the conduction within the left bundle branch [4,5]. In addition to its use in atrial fibrillation, flecainide can also be used to suppress isolated premature ventricular contraction and non-sustained ventricular arrhythmia [6].

As seen with the patient presented above, flecainide use is not without its side effects. Its toxicity level has been associated with the plasma level of the drug [7]. Flecainide is a drug that is renally excreted; as a result, any condition that can result in renal dysfunction can lead to elevated levels of flecainide within the plasma [8]. Over the course of her hospital stay, our patient developed an acute kidney injury which caused an increase in the plasma concentration of flecainide in the blood. Unmonitored toxicity can cause ventricular dyssynchrony or fatal arrhythmias. Flecainide toxicity can present with wide or sinusoidal QRS complexes due to sodium channel toxicity [3]. Flecainide decreases the electrical conduction in the left ventricular myocardium through its inhibition of the inward sodium current which results in ventricular dyssynchrony and LBBB at toxic levels. Additionally, widening of the QRS has been shown to be related to reduced left ventricular contractility [9]. Generally, discontinuation of the agent will result in the resolution of the LBBB. 

## Conclusions

Although flecainide is one of the first-line antiarrhythmic agents in patients without structural heart disease, caution should be exercised with its use. Obtaining a baseline EKG prior to initiation of the drug is important to assess baseline intervals. Additionally, echocardiography and further workup for ischemic diseases should be considered if systolic dysfunction is discovered to prevent worsening left ventricular contractility that can result from the use of flecainide. 
